# Adiponectin, Obesity, and Cancer: Clash of the Bigwigs in Health and Disease

**DOI:** 10.3390/ijms20102519

**Published:** 2019-05-22

**Authors:** Sheetal Parida, Sumit Siddharth, Dipali Sharma

**Affiliations:** Department of Oncology, Johns Hopkins University School of Medicine and the Sidney Kimmel Comprehensive Cancer Center at Johns Hopkins, Baltimore, MD 21231, USA

**Keywords:** adiponectin, obesity, cancer

## Abstract

Adiponectin is one of the most important adipocytokines secreted by adipocytes and is called a “guardian angel adipocytokine” owing to its unique biological functions. Adiponectin inversely correlates with body fat mass and visceral adiposity. Identified independently by four different research groups, adiponectin has multiple names; Acrp30, apM1, GBP28, and AdipoQ. Adiponectin mediates its biological functions via three known receptors, AdipoR1, AdipoR2, and T-cadherin, which are distributed throughout the body. Biological functions of adiponectin are multifold ranging from anti-diabetic, anti-atherogenic, anti-inflammatory to anti-cancer. Lower adiponectin levels have been associated with metabolic syndrome, type 2 diabetes, insulin resistance, cardiovascular diseases, and hypertension. A plethora of experimental evidence supports the role of obesity and increased adiposity in multiple cancers including breast, liver, pancreatic, prostrate, ovarian, and colorectal cancers. Obesity mediates its effect on cancer progression via dysregulation of adipocytokines including increased production of oncogenic adipokine leptin along with decreased production of adiponectin. Multiple studies have shown the protective role of adiponectin in obesity-associated diseases and cancer. Adiponectin modulates multiple signaling pathways to exert its physiological and protective functions. Many studies over the years have shown the beneficial effect of adiponectin in cancer regression and put forth various innovative ways to increase adiponectin levels.

## 1. Discovery of a Guardian Angel Adipocytokine

White adipose tissue (WAT), once regarded as the major site of energy storage and homeostasis, is now known to be an endocrine organ producing numerous biologically active molecules and hormones, one of the most important being adiponectin. Mainly secreted by adipocytes, adiponectin is also produced to some extent by bone marrow, osteoblasts, fetal tissue, myocytes, cardiomyocytes, and salivary gland epithelial cells [1,2]. The first report on adiponectin was published in 1995, where it was denoted as an adipocyte complement related protein of 30 kDa (Acrp30), specifically expressed in the adipose tissues and differentiated adipocytes [3]. Another group identified mouse adiponectin and referred to it as AdipoQ using an mRNA differential display technique. They reported 247 amino acids polypeptide coded by adipoQ cDNA specifically in adipose tissues of mice and rats. Importantly, they also showed the reduction of adipoQ mRNA in obese mice and humans [4]. These two pioneering papers indicated the function of adiponectin in energy homeostasis. The next few years observed a revolutionary rise in the exploration of adiponectin. In 1996, another research group discerned adiponectin as the most abundant transcript in the cDNA library of human adipose tissue, which was termed as adipose most abundant gene transcript1 (*apM1*) [5]. In the same year, yet another group isolated adiponectin from human plasma using affinity chromatography, followed by protein sequencing and referred to as gelatin-binding protein of 28 kDa (GBP28) [6]. Adiponectin is a small protein composed of 224 amino acids, present in circulating concentrations as high as 2 to 10 μg/mL in humans. The protein encompasses a signal domain followed by a variable domain which is species specific, a collagen domain, and a globular domain. 

Berg and colleagues and Yamauchi and colleagues were the first to identify the physiological importance of adiponectin and highlighted the adiponectin axis as a possible therapeutic field for the treatment of diabetes [7,8]. Enhanced circulating levels of adiponectin inhibits gluconeogenesis [9]. They concluded that reduced adiponectin in obese- and adipose-tissue-deficient mice serve as a responsible factor for the development of insulin resistance [9]. The work of Yamauchi et al. [8] reported decreased adiponectin in insulin resistance and altered insulin-sensitive mice models. In the continuation work, Yamauchi et al. showed that in skeletal muscle, both globular as well as full-length adiponectin are able to induce AMP activated protein kinase (AMPK), while only full-length adiponectin can stimulate AMPK in liver cells [10]. Ahima and co-workers [11] demonstrated that intravenous injections of adiponectin lead to an increase in the adiponectin level in cerebrospinal fluid. Intracerebroventricular administration of adiponectin in leptin-induced obese mice caused enhanced thermogenesis, weight loss, and decrease in serum glucose and serum lipid levels [11]. The second decade of the 21st century observed the rediscovery of the physiological role of adiponectin. Holland et al. [12] observed increased ceramide content in the liver of obese mice (ob/ob mice or high-fat diet mice). An increase in ceramide is associated with insulin resistance, cell death, and atherosclerosis [13]. Holland et al. [12] reported that adiponectin enhances the ceramide catabolism in the liver via the ceramidase activity of its receptors, AdipoR1 and AdipoR2, which was independent of AMPK activation. Xia et al. reported a decrease in the ceramide level in transgenic mice with genetically induced acid ceramidase activity in the hepatic cells or in adipose tissues [14]. The contribution of adiponectin is not only restricted to liver, but also extends to other major organs. Rutkowski et al. showed that overexpression of adiponectin recovered the kidney podocytes rapidly and demonstrated low intestinal fibrosis. But the lack of adiponectin caused irreparable albuminuria and damage in kidney podocytes [15]. Adiponectin enhances the myocyte enhancer factor-2 (MEF2) induction in cardiomyocytes via p38 MAPK (mitogen-activated protein kinases) signaling [16]. 

Adiponectin works by AdipoR1 and AdipoR2 receptors, which are unique and universally expressed. AdipoR1 is most abundantly found in skeletal muscle whereas AdipoR2 is predominantly present in liver [17]. AdipoR1 exhibits higher affinity for globular adiponectin, whereas AdipoR2 shows higher affinity for full-length adiponectin [17]. Both AdipoR1 and AdipoR2 accumulate in homodimeric and heterodimeric complexes once bound by adiponectin [17]. Hug et al. [18] identified T-cadherin, a member of the cadherin superfamily, as an effective receptor of hexamers and of high molecular weight (HMW) adiponectin oligomers. Adiponectin can exert its biological functions by directly interacting with its specific receptors that provide some organ and functional specificity to adiponectin. In addition, multiple regulatory mechanisms tightly regulate adiponectin and further control its biological impact on various organs in normal as well as disease state. 

## 2. Tight Regulation of Adiponectin at Multiple Levels

### 2.1. The Role of Coactivators in Transcriptional Regulation of Adiponectin

Peroxisome proliferator-activated receptor gamma is an important transcription factor belonging to the PPAR family, is a positive regulator of adiponectin transcription, and is widely expressed in adipose tissue. Lower adiponectin levels have been associated with the P12A mutation in PPARγ. The Thiazolidinedione class of medications (TZDs) are PPARγ agonists that stimulate adiponectin production and are used as antidiabetics. Its efficacy has been shown in vitro as well as in vivo studies [19,20,21] but could be limited by a mutation at a putative PPARγ-recognizing PPAR response element (PPRE) site [19]. However, some reports demonstrate an increase in high molecular weight (HMW) adiponectin biosynthesis in response to TZDs without changing adiponectin mRNA levels indicating a predominantly translational regulation of adiponectin synthesis. Forkhead box protein O1 (FoxO1) is another key regulator of adipocyte differentiation which is known to positively regulate adiponectin transcription [22]. Biological functions and cellular localization of this transcription factor is regulated by NAD-dependent deacetylase Sirt1 (sirtuin 1) and by additional post-translational modifications including phosphorylation and acetylation. It complexes with CCAAT-enhancer-binding proteins (C/EBPα) which in turn is stimulated by Sirt1 overexpression, consequently, activating adiponectin promoter [23]. However, FoxO1 activity could be controlled by multiple upstream events that can in turn regulate adiponectin levels. C/EBPα interacts with the CCAAT motif of the adiponectin promoter recruiting co-activators, in turn stimulating transcriptional activity. Co-expression of PPARγ and C/EBPα are known to significantly increase adiponectin expression [24,25]. Sterol regulatory element-binding proteins (SERBPs) are membrane-bound precursors that interact with the nuclear envelope or ER (endoplasmic reticulum) membranes. Sterol regulatory element-binding proteins regulate the transcription of lipid-metabolizing enzymes. Binding of SREBP to the SERBP response element (SRE) on adiponectin promoter amplifies adiponectin expression. Adiponectin promoter is also transactivated by SREBP-1c and is prevented in case there is a mutation in the SRE motif. Adenovirus-mediated overexpression of SREBP-1c is known to elevate adiponectin levels in 3T3-L1 adipocytes [26]. 

### 2.2. Involvement of Multiple Co-Factors in Transcriptional Repression of Adiponectin 

cAMP response element-binding protein (CREB) is a master regulator of adipogenesis and has been associated with systemic insulin resistance in obese state [27]. Camp response element-binding protein indirectly represses adiponectin transcription by upregulating transcription factor ATF3 (activating transcription factor 3), which binds to the AP-1 (activator protein-1) site next to the NFAT (nuclear factor of activated T-cells) binding site of adiponectin promoter [28]. Nuclear factor of activated T-cell proteins are calcium-sensitive proteins associated with immune functions and have been detected in 3T3-L1 adipocytes [29]. Overexpressed in obesity and diabetes, they have been associated with WAT activity but the exact mechanism of NFAT in adiponectin regulation is unclear. Nuclear factor of activated T-cell binding site deletion in adiponectin promoter [27] enhances adiponectin expression while overexpression of NFAT diminishes adiponectin transcription. Additional transcription factors involved in downregulation of adiponectin transcription include AP-2β (activating enhancer binding protein-2β), IGFBP-3 (IGF-1-binding protein 3), and Id3 (inhibitor of differentiation-3) [30]. Fat accumulation in obese state induces a hypoxic microenvironment, which is known to inhibit adiponectin transcription via hypoxia inducible factor 1 alpha (HIF1α). Obesity-induced chronic inflammation leads to overexpression of TNFα (tumor necrosis factor alpha), IL6 (interleukin 6), IL18 (interleukin 18), and other pro-inflammatory cytokines that are also known to inhibit adiponectin. Tumor necrosis factor alpha suppresses transcription activator PPARγ via JNK (c-Jun N-terminal kinases)-mediated phosphorylation which reduces its DNA binding [31]. Tumor necrosis factor alpha also promotes IGFBP-3 inhibiting adiponectin transcription and conferring insulin resistance [32]. Tumor necrosis factor alpha has also been shown to inhibit FoxO1 and C/EBPα [30,32]. Interleukin 6 suppresses adiponectin transcription in 3T3-L1 adipocytes via p44/42 MAPK pathway [33]. Interleukin 18, on the other hand, phosphorylates and activates NFATc4, a repressor of adiponectin transcription in a ERK (extracellular-signal-regulated kinase) 1/2 dependent manner [28]. 

### 2.3. Control of Adiponectin Expression via Post-Translational Modifications

**Figure 1:**
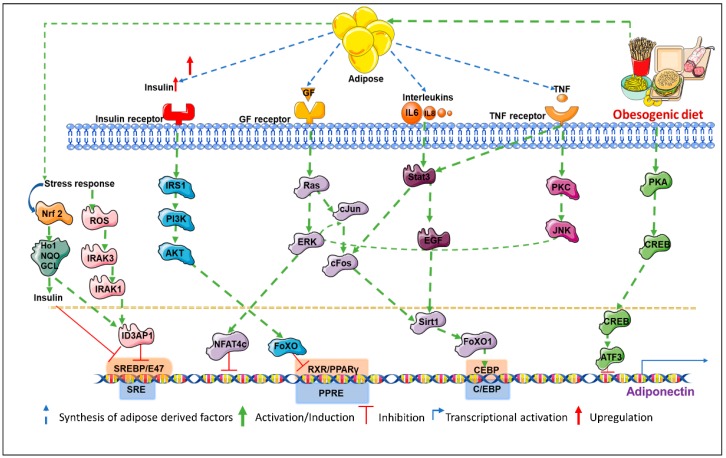
Multiple signaling networks converge to regulate adiponectin.

Post-translational modifications are the most important determinants of adiponectin functionality since different isoforms (trimeric, hexameric, and HMW multimeric forms) of adiponectin exhibit different biological activities. While trimeric and hexameric forms mostly regulate food intake [34], HMW forms of adiponectin mostly regulate insulin sensitivity, hepatic gluconeogenesis, and other metabolic functions [35]. Other forms also mediate some metabolic functions. Since different forms of adiponectin function differently, activity of adiponectin can be modulated by changing the ratios of different isoforms of adiponectin in serum. For example, activity of thiazolidinediones, statins, and angiotensin receptor blockers depend on increasing the proportion of HMW adiponectin [30]. There is very little evidence to support the notion that adiponectin isoforms can interconvert after secretion indicating that modulation of intracellular processes guiding multimerization of adiponectin is important. Multimerization of adiponectin into HMW complexes is a complicated and active area of research. Structurally, adiponectin has an N-terminal variable domain, a collagenous domain, and a C-terminal globular domain. Production and secretion of HMW adiponectin is dependent on hydroxylation and glycosylation of lysine residues of the collagenous domain [36,37,38,39]. Substitution of lysine residues for arginine completely abolishes HMW adiponectin synthesis. Multimerization also requires proline hydroxylation [39]. Inhibiting prolyl and lysyl hydroxylases using 2,20-dipyridyl can completely impair adiponectin multimerization [39]. ER retention of folded adiponectin molecules without being secreted leads to ER stress. It is very common in obese state and it is a major factor causing low circulating adiponectin levels. ER retention of adiponectin is maintained by thiol-mediated retention via (ER) chaperone protein 44 (ERp44) [40]. Thiol bond-reducing agents induce a 7- to 8-fold increase in secretion of adiponectin in 3T3-L1 adipocytes. Thiol-bond formation is also the determinant of adiponectin folding and assembly prior to secretion [40]. Another important factor in multimerization and release is intermolecular disulfide bond exchange by ER chaperone Ero-1La [41]. Increased expression of Ero-1La or inhibition of SIRT1, suppressor of Ero-1La results in increased assembly and release of HMW adiponectin [41]. Similarly, ERp44 inhibition using TZDs may be utilized to prevent ER retention of adiponectin and increase its circulatory levels [42]. DsbA-L (disulfide-bond A oxidoreductase-like protein) is yet another ER chaperone known to directly bind to adiponectin and aid HMW multimerization [43]. Though the mechanism is not completely understood, PPARα agonists are known to upregulate DsbA-L [43]. Hence, a combination of molecular events at the transcriptional and translational level regulate adiponectin (Figure 1).

## 3. Physiological Functions of Adiponectin 

### 3.1. A Central Role in the Reproductive System

Similar to other adipokines like leptin, resistin, and ghrelin, adiponectin plays a central role in reproductive functions. Circulating levels of adiponectin are known to be higher in females compared to males; testosterone exposure has been shown to reduce serum adiponectin levels. In the ovaries, adiponectin receptors 1 and 2 have been found to be expressed in granulosa cells, oocytes, and corpus luteum [44]. Adiponectin induces expression of cyclooxygenase 2 (COX2), vascular endothelial growth factor (VEGF), and prostaglandin E synthase (PGES) in granulosa cells in a porcine model [1]. Similarly, increased adiponectin and *AdipoR1* gene expression was observed in immature rat ovaries in response to human chorionic gonadotropin (hCG) [45]. Some studies also suggest a role of adiponectin in ovarian steroidogenesis, while additional studies showed species-specific variable results. Gene expression changes induced by adiponectin in the ovaries have been demonstrated to be modulated by AMPK (AMP activated protein kinase) or ERK1/2-MAPK dependent pathway [45]. Adiponectin-induced AMPK has been shown to regulate the energy requirement for follicular growth. AMPK phosphorylates PPARγ, which structurally resembles steroid hormone receptors, repressing its transactivation [45]. PPARγ is known to influence steroidogenesis, ovulation, oocyte maturation, and maintenance of corpus luteum [45]. AMPK and PPARγ, therefore, cooperatively regulate the energy balance in the ovary, thus, ensuring optimum growth of ovarian follicles [46,47]. However, mice lacking functional AdipoR1 or AdipoR2 do not demonstrate defective reproduction suggesting that adiponectin is not indispensable for reproductive functions and evidence suggests adiponectin’s effect on the ovaries is by virtue of its insulin sensitizing efficiency [46,47]. The essential role of adiponectin has been explained in the early stages of fetal development [48]. Adiponectin receptor expression is higher in the endometrial epithelium of women in the mid-secretory phase of the menstrual cycle indicating a role of adiponectin in the endometrial changes associated with embryo implantation [49]. Additionally, adiponectin inhibits IL-1β-mediated inflammatory response via AMPK [49] in the stromal cells of endometrium. Adiponectin has also been detected in early developmental stages of rabbits, pigs, and mice embryos, and improves development of pig embryo by accelerating meiosis in a p38MAPK dependent manner. During developmental stages, adiponectin is not confined to adipose tissue but is also expressed in epidermis, smooth muscle fibers, small intestine wall, major arterial vessels, and ocular lens suggesting multifold functions of the hormone that remain to be understood. Gestational diabetes, a common pregnancy-related complication is also correlated with plasma adiponectin levels. Women who develop gestational diabetes during late pregnancy exhibit lower adiponectin levels in early pregnancy [1]. Hypoadiponectinemia has also been associated with polycystic ovary syndrome (PCOS), a major cause of anovulatory infertility, though reports have been inconsistent. Polycystic ovary syndrome is known to have a strong genetic association and it has been reported that single nucleotide polymorphism (SNP) in the adiponectin gene might be associated with increased risk of PCOS. Polycystic ovary syndrome patients are susceptible to glucose intolerance, insulin resistance, hypertension, and hyperlipidemia with evidently low circulating adiponectin levels [1]. 

### 3.2. Regulation of Insulin Sensitivity and Protection against Fatty Liver

Adiponectin is shown to be protective against fatty liver disease and a low circulating adiponectin has been observed in patients with chronic hepatitis and liver steatosis; inverse quantitative correlation between circulating adiponectin and grade of hepatic steatosis has been found. Some studies also suggest a SNP variation in adiponectin and mutation in AdipoR2 receptor to be associated with hepatic steatosis and fibrosis [50,51]. The most important known biological role of adiponectin is the regulation of insulin sensitivity in muscle cells, which makes it a central player in type 2 diabetes mellitus (T2DM) and metabolic syndrome. In humans, adiponectin is also known to be secreted by the skeletal muscles where it regulates lipid metabolism via AMPK, p38MAPK, and PPARα pathways [52,53] resulting in more efficient glucose metabolism via glucose transporter type 4 (GLUT4) receptor and fatty acid oxidation, thus maintaining insulin sensitivity [54,55]. Though both AdipoR1 and AdipoR2 have been detected in the skeletal muscles, the relative levels of the two receptors appear to be regulated by insulin levels, fasting–feeding cycles, and other pathophysiological situations; potentially via a PI3K/FoxO1 mediated pathway [56]. Direct role of adiponectin in regulating insulin sensitivity and the fact that adiponectin deficient mice are insulin resistant accompanied by lower insulin production in response to glucose intake suggests a potential role of adiponectin in regulation of insulin production by the β-cells which express both AdipoR1 and AdipoR2 [57]. Adiponectin receptor levels are also lower in pancreas of genetically obese mice. Consistently, adiponectin administration increases insulin secretion in response to glucose in experimental mice [58,59]. In vitro, adiponectin has been shown to prevent free fatty acid induced programed cell death in β-cell lines [59]. Adiponectin, thus, plays an important role in survival and functions of the pancreas protecting it from physiological damage, which in turn regulates the levels of insulin in the body that is central to most metabolic processes. In fact, Zyromski et al. [60] elegantly demonstrated that increasing circulating adiponectin in obese rodents by cannabinoid receptor-1 antagonist leads to recovery of acute pancreatitis.

### 3.3. Adiponectin in the Central Nervous System

Adiponectin receptors have been detected throughout the central nervous system including the hypothalamus and brainstem and can stimulate neuroendocrine and autonomic responses in the central nervous system (CNS) [61]. While some studies suggest that intracerebroventricular administration of adiponectin results in elevated energy expenditure and promotes weight loss without effecting appetite, some other studies show adiponectin induces increased food intake and reduced energy expenditure. While these results are conflicting, it also draws attention to the fact that adipokines by nature are neuromodulators that signal the brain to regulate food intake and energy expenditure in a context specific manner. Adiponectin receptors have been detected in the pituitary gland of humans suggesting a direct role in regulation of pituitary functions. Using in vitro models, adiponectin has been demonstrated to inhibit growth hormone (GH) and luteinizing hormone (LH) production by rat pituitary cells [62,63]. Yet other studies have indicated a cerebro-protective role of adiponectin via endothelial nitric oxide synthase (eNOS)-mediated pathway as well as migraine-associated inflammation and vasodilation, though more detailed studies are required [63]. In addition to playing important roles in normal physiology, an imbalance in adiponectin levels is associated with multiple pathophysiological states. 

## 4. Perturbations in Adiponectin Levels Manifest in Disease States

### 4.1. Association of Adiponectin with Inflammation

In contrast to most other adipokines, adiponectin is a well-known anti-inflammatory agent. Using in vitro systems, it has been demonstrated to inhibit B cells differentiation from bone marrow and elevate expression of anti-inflammatory cytokines IL10, IL6, TNFα, and IFNγ in monocyte-derived cell types by inhibiting NFκB (nuclear factor kappa light-chain-enhancer of activated B cells) pathway [64,65,66]. However, the response is variable depending on the isoforms of the hormone and the cell type targeted, for example, while low molecular weight adiponectin can inhibit LPS-induced IL6 and elevate IL10 in differentiated TH1 macrophages, the multimers of the adipokine do not modulate the abovementioned cytokines [64]. The HMW adiponectin elicit IL6 secretion from monocytes and THP-1 macrophages [64]. Reciprocal regulation of adiponectin by pro-inflammatory cytokines is equally well known. Obesity is a chronic state of low-grade inflammation with sustained and significantly higher level of inflammatory cytokines like TNFα that is known to directly inhibit adiponectin transcription. A large body of research suggests that a higher level of circulating adiponectin in lean humans induces a resistance to inflammatory stimulus in the macrophages. The context specific role of adiponectin in inflammatory processes is also implicated by the fact that higher levels of adiponectin in serum and synovial fluid of rheumatoid arthritis (RA) patients where a sustained inflammatory environment results in degradation of joints and disease severity is directly proportional to circulating adiponectin levels [67,68]. Both adiponectin and adiponectin receptors have been detected in synovial fibroblast of RA patients indicating a local paracrine signaling event [67,68]. In these synovial fibroblasts, adiponectin induces IL6 and pro-matrix metalloproteinase-1 (pro-MMP1) synthesis via p38MAPK and NFκB pathways without influencing other cytokines [69]. It has also been reported to escalate IL8 production which can be attenuated by inhibiting AdipoR2 using RNA interference but not via AdipoR1 [69].

### 4.2. Adiponectin in Cardiovascular Diseases

Cardiovascular diseases (CVDs) strongly correlate with visceral adiposity which in turn associates with lower circulating adiponectin, verifiable in patients with cardiovascular disease [70]. Similar to other physiological scenarios, CVD dependence on adiponectin levels is context specific. Lower levels of HMW adiponectin correlate with incidence of CVD [71]. Hypoadiponectinemia has been associated with severity of myocardial infraction with elevated levels of TNFα and increased apoptotic death in myocytes and stromal cells [72]; adiponectin administration attenuates these complications and reduces severity of infraction via the COX-2/EP4 pathway mediated by AMPK [73]. Adiponectin regulates the cardioprotective COX-2 signaling via sphingosine kinase (SphK) signaling [73,74]. Additionally, adiponectin has also been studied in the context of atherosclerosis. In injured blood vessels, adiponectin has been reported to bind collagen type I, III, and V, suggesting a role in repair of vasculature [1]. It suppresses vascular cell adhesion protein-1 (VCAM-1) expression in monocytes resulting in suppression of TNFα synthesis, thus preventing them from adhering to the aortic endothelial cells [75]. Adiponectin also inhibits the class A macrophage scavenger receptor in macrophages preventing them from being converted into foam cells, the culprits of atherosclerosis. It has also been proposed that adiponectin gene variation can be used as a predictor of coronary heart disease risk since T/T homozygotes of the adiponectin gene were at lower risk of developing coronary artery disease compared to G/G or G/T genotype individuals [50].

## 5. Obese State and Adiponectin—An Inverse Relation

In contrast to other known adipokines, adiponectin is inversely related to body mass index (BMI) and central adiposity; the strongest negative correlation has been observed with waist-to-hip ratio [76]. Undoubtedly, a feedback loop regulates it at transcriptional, translational or post-translational level. A similar trend in downregulation of its receptors AdipoR1 and AdipoR2 have also been observed [77]. Normal levels of adiponectin as well as its receptors are re-established post weight/fat loss. Though a number of mechanisms have been proposed, none of them precisely explain the feedback mechanism in adiponectin regulation. An obese state is a situation of chronic inflammation in the body characterized by a marked increase in the levels of inflammatory cytokines IL6, IL8, TNFα, and leptin, which are directly known to inhibit adiponectin transcription. In addition, the most important and well-understood role of adiponectin is insulin sensitization in the skeletal muscles. Increase in visceral fat mass lowers systemic adiponectin levels creating insulin resistance in the skeletal muscle while glucose signaling in response to food intake stimulates elevated insulin secretion by the pancreas that is free from adiponectin’s regulatory control due to its lower circulating concentrations. As a result, there is increased conversion of glucose and glycogen into fats, which is then taken up by the skeletal muscles leading to intramuscular fat accumulation, typical of type 2 diabetes. Consequently, a vicious cycle is initiated where increased adiposity causes a drop in adiponectin levels that in turn results in fat accumulation in muscles and vital organs like liver that further lowers adiponectin levels giving way to cardiovascular diseases and atherosclerosis. Again, gender specific differences are evident since lower adiponectin levels have been recorded in diabetic men compared to women which has been partially attributed to higher testosterone levels in men.

Though both total and HMW adiponectin are downregulated, HMW adiponectin is a better predictor of insulin resistance [78,79]. As such, individuals harboring mutations for adiponectin multimerization are more susceptible to type II diabetes [78]. Lipoatrophic mice lacking circulating adiponectin were found to be hyperglycemic as well as had higher levels of insulin, both of which could be reverted by continuous adiponectin administration. In light of genetic association, +45 G-allele of the adiponectin gene has been shown to regulate glucose tolerance and insulin sensitivity [50,80]. Similarly, AdipoR1 −3882 T > C polymorphism has been shown to be responsible for lower insulin resistance and fasting glucose levels [50,80]. Adiponectin has been shown to have a direct and immediate effect on blood pressure and lower circulating adiponectin levels can be considered a predictor of hypertension risk. Experimentally, angiotensin II administration decreases circulatory adiponectin while angiotensin II receptor blockade results in increase in its plasma concentrations [81,82]. Adiponectin has been widely studied in the context of lipoprotein metabolism, a dysregulation of which is termed as dyslipidemia. Obesity is often characterized by increased triglycerides, free fatty acids and low-density lipoprotein (LDL), and a decrease in high-density lipoprotein (HDL). Circulatory adiponectin positively correlates with HDL and size density of LDL, and negatively correlates with plasma triglycerides [83,84]. Hypoadiponectinemia often associates with an atherosclerotic lipid profile. The positive correlation between HDL and adiponectin is known to be regulated by apolipoprotein A-I (apoA-I) [85]. Metabolic syndrome is defined as the physiological state of complete metabolic dysfunction characterized by hyperglycemia, insulin resistance, hypertension, dyslipidemia, and obesity. All these conditions are strongly associated with lower adiponectin levels, particularly, HMW adiponectin [86,87]. It has also been shown to be a predictor of metabolic syndrome in a 6-year follow-up study in Japan [87]. Though few studies have been successfully conducted, the physiological relevance of adiponectin in eating disorders like bulimia and anorexia nervosa is still unclear, since both upregulation and downregulation of the adipokines in these conditions have been reported. More detailed analyses with special attention to the confounding factors is required to clearly delineate the inverse relationship between obesity and adiponectin. 

## 6. Adiponectin- and Obesity-Associated Disorders

Deregulated adiponectin production in obesity may be the leading cause of endometrial impairment, hypertension, myocardial infarction, and other complexities of metabolic syndrome along with cancer initiation and progression (Table 1). 

### 6.1. Hypertension

Several factors contribute to the association of obesity and hypertension including sympathetic activation of the nervous system, endothelial dysfunction (due to an increase in free fatty acids and oxidative stress), and an abnormal adipokine production [88]. Adults with hypertension display lower levels of adiponectin [89]. Total adiponectin levels were found to be lower in obese individuals suffering from hypertension in comparison to lean and normotensive individuals [90]. Adiponectin coordinates blood pressure by mechanisms regulated by brain and endothelium [91,92]. Studies reveal that adiponectin suppresses TNFα and inhibits foam cell transformation of macrophages [93]. Adiponectin prevents the atheroma formation by nitric oxide (NO) production via phosphoinositide 3-kinase (PI3K) and AMPK pathways in endothelial cells [91,92]. Adiponectin also reduces the proliferation of smooth muscle cells and TNF-α in macrophages [93].

### 6.2. Atherosclerosis

There are multiple mechanisms linking obesity to cardiovascular diseases [94,95]. Several adipokines facilitate the cross-talk between adipose tissue, heart, and vessels in the “adipo-cardiovascular axis”. A prothrombotic state is stimulated by the altered release of adipokines that leads to cardiovascular disease and atherosclerosis [96,97]. Reduced levels of serum adiponectin are interpreters of atherosclerosis and myocardial infarction. Additionally, there is a strong correlation between hypoadiponectinemia and coronary heart diseases well supported by clinical trials which confirm that higher incidences of cardiovascular events are associated with lower levels of adiponectin [98]. Reports suggest HMW adiponectin to be a better independent risk factor than the total adiponectin for cardiovascular diseases [99,100]. In vivo studies using adiponectin deficient mice reveal severely injured arteries while adiponectin supplementation impaired neointimal proliferation. In vitro culture studies demonstrate that platelet-derived growth factor (PDGF), heparin-binding epidermal growth factor (HB-EGF), basic fibroblast growth factor (BFGF), and epidermal growth factor (EGF)-induced DNA synthesis, cell proliferation and cell migration are impaired by adiponectin [101]. Adiponectin reduces inflammatory cytokines and adhesion molecules in endothelial cells. Apart from inhibiting the conversion of macrophages into foam cells, adiponectin also decreases TNF-alpha production as well as induces the production of the anti-inflammatory cytokine, IL-10 [102].

### 6.3. Obstructive Sleep Apnea Syndrome

Obstructive sleep apnea syndrome (OSAS) is a condition characterized by recurrent respiratory disorders during sleep [103]. The level of adiponectin is undoubtedly lower in OSAS patients. A study by Hargens et al. [104] confirmed lower adiponectin levels in OSAS patients in comparison to controls. Yet, there are some studies which suggest that patients suffering from OSAS do not show alteration in adiponectin levels. Intermittent hypoxia resulting in a decrease of total and HMW adiponectin is argued to be the major cause of the reduction of adiponectin in OSAS [105,106].

### 6.4. Diabetic Retinopathy

One of the major risk factors for diabetic microvascular complications is obesity. Increased levels of glucose in T2DM are thought to be a risk for microvascular (retinopathy, nephropathy and neuropathy) and macrovascular (coronary heart disease, stroke and peripheral vascular disease) complications [107]. The most common complication of diabetic microvascular disease is diabetic retinopathy which affects 30–50% of all diabetics [107]. Both obesity and T2DM patients display decreased adiponectin levels in circulation. Additionally, T2DM patients with diabetic retinopathy (non-proliferative and proliferative) have reduced levels of adiponectin compared to patients without retinopathy [108].

## 7. Obesity, Adiponectin, and Cancer: Interplay of Bigwigs

Multiple epidemiological evidences associate obesity with the risk of cancer development. The study conducted by the American Cancer Society comparing individuals with a body mass index (BMI) over 30 kg/m^2^ with individuals over 25 kg/m^2^ concluded that the relative risk of colorectal cancer is at 1.8 for obese males and 1.2 for obese females [109]. A meta-analysis of 11 studies indicated the probability of 6% increase in the risk of kidney cancer in men and 7% in women per unit BMI increase with an average 36% higher risk in overweight individuals (BMI > 25 kg/m^2^) and 84% higher risk in obese individuals (BMI > 30 kg/m^2^) [109]. Lagergren et al. reported a positive correlation of esophageal carcinoma with increased BMI (>25.6 kg/m^2^ in males and >24.2 kg/m^2^ in females) along with a higher risk in individuals with a BMI greater than 30 kg/m^2^ [109]. According to the International Agency for Research on Cancer and the World Cancer Research Fund (WCRF), obesity is strongly associated with endometrial cancer, adenocarcinoma of the esophagus, colorectal cancer, postmenopausal breast, prostate, and renal cancer. Leukemia, non-Hodgkin’s lymphoma, multiple myeloma, malignant melanoma, and thyroid tumors represent some lesser common malignancies associated with obesity [89,110,111]. The strongest correlation between obesity and cancer risk has been observed in the case of breast cancer. Approximately 80% of the breast is composed of adipose tissue or fat. The mammary epithelial cells are therefore in close contact with a cocktail of adipokines produced by the adipose tissue and any imbalance in the hormonal milieu renders the breast susceptible to tumorigenesis. Reduced levels of total and HMW adiponectin have been shown be associated with breast cancers irrespective of age, BMI, hormone status, and other factors which was first reported by Noguchi and group [112,113]. Adiponectin and its receptors are known to be expressed in breast epithelial as well as myoepithelial cells of the breast. Cytoplasmic expression of both the AdiopRs is known in normal breast epithelial and breast cancer cells but breast cancer tissue exhibits a higher expression of AdipoR2 which also significantly and positively correlates with vascular and lymphovascular invasion in breast cancer. Adiponectin receptors are known to be expressed on most breast cancer cell lines including MCF7, T47D, MDA-MB-231, MDA-MB-361, and SKBR3. While MDA-MB-231, T47D, and MCF-7 showed higher expression of AdipoR1, MDA-MB-361 had higher expression of AdipoR2. Adiponectin protein distribution also varied between cell lines. Insulin, insulin-like growth factor-1 (IGF1), leptin, adiponectin, steroid hormones, and cytokines are some host factors associated with obesity that not only influences the initiation and progression of breast cancer, but also affects its response to therapies [114]. Reports reveal decreased adiponectin in breast and endometrial cancer and vice versa in non-small cell lung cancer, pancreatic, liver, prostate, gastric, renal cell carcinoma, and colon cancer [115,116,117,118]. Wei et al. [119] performed a meta-analysis of 107 studies in a random effect model to analyze the levels of circulating adiponectin in cancer patients versus controls. They found that the circulating adiponectin levels were significantly downregulated in cancer patients compared to control patients with a pooled SMD of −0.334 μg/mL. Further analysis of eight different studies showed that the circulating levels of HMW adiponectin was lower in cancer cases than control cases with a pooled standard mean differences (SMD) of −0.502 μg/mL (Table 2). 

## 8. Adiponectin Orchestrates Multiple Biological Functions to Inhibit Cancer Progression 

### 8.1. Inhibition of Angiogenesis

Tumor growth and metastasis are high-energy expenditure processes requiring constant supply of growth factors and nutrients, which is ensured by profusely leaky tumor vasculature. Adipocytes as well as pre-adipocytes are known to synthesize proangiogenic factors including leptin, TNFα, IL6, HGF (hepatocyte growth factor), and bFGF. Vasculogenesis requires fibroblast growth factor-2 (FGF-2) for proliferation of endothelial cells followed by migration of the endothelial cells and tubulogenesis that is facilitated by the vascular endothelial growth factor, VEGF. Using culture-based studies, it has been demonstrated that adiponectin is capable of inhibiting endothelial cell proliferation induced by FGF2 as well as migration of endothelial cells by VEGF. In a mouse fibrocarcinomas model, intratumoral administration of adiponectin resulted in disruption of tumor vasculature and caspase-3-mediated intratumoral apoptosis, possibly by nutrient deprivation of tumor cells, resulting in over 60% tumor regression. Adiponectin inhibits in vitro proliferation of human umbilical vein endothelial cells (HUVECs) and subsequent vessel formation. Adiponectin null mice have also been shown to exhibit retarded tumor growth, diminished vascularization, and inhibition of pulmonary metastasis [128]. However, contradictory results have also been observed in literature and regulation of vasulogenesis by adiponectin warrants further investigation. 

### 8.2. Inhibition of Growth and Proliferation

Adiponectin has been shown to inhibit cell proliferation via ERK1/2-MAPK pathway in T47D cells. In MDA-MB-231 xenografts, recombinant as well as adenovirus-mediated adiponectin overexpression, regressed tumor development, inhibited secondary tumor development in adjacent fat pads, and prevented lung metastasis via the GSK3β/βcatenin signaling pathway [113,129]. Kang et al. [130] reported that inhibitory effects (growth arrest as well as apoptosis) of adiponectin have been more pronounced in the mesenchymal-like cell line MDA-MB-231 compared to the luminal-like cell line T47D by inducing G0/G1 cell cycle arrest. Adiponectin is thought to enhance Bax and p53 expression while downregulating CyclinD1, blocking JNK signaling, and inducing PARP cleavage via AdipoRs [113] Another proposed mechanism of adiponectin-induced growth inhibition and apoptosis in MCF7, T47D, SK-BR3, and MCF10A is AMPK activation as a result of adiponectin binding to AdipoRs resulting in p42/p44MAPK inhibition which consequently modulates p53, Bax, Bcl-2, c-myc, and cyclin D1 [113]. Grossman et al. further explain that estrogen receptor alpha (ERα) positive cell lines MCF7 and T47D cells could be inhibited even with low concentrations of adiponectin, but to achieve similar results in ERα negative cell line SKBR3, much higher concentrations of the adipokines are necessary. They further investigated the role of ER in adiponectin-mediated inhibition by expressing estrogen receptor in MDA-MB-231 which resulted in cells being responsive to adiponectin-induced growth inhibition via a blockade of JNK2 signaling [113]. Adiponectin has also been shown to inhibit leptin-induced cell proliferation in MDA-MB-231, MDA-MB-361, SK-BR-3, MCF-7, and T47D cells at varying doses [120]. Genetic variations in adiponectin and its receptor has also been suggested to be associated with breast cancer risk. One study found that the women who had adiponectin rs2241766 (+45 T → G) TG genotype, the genotype associated with higher circulatory adiponectin, were at 39% lower risk of developing breast cancer where as those with adiponectin rs1501299 (+276 G → T) TG and GG genotypes, low adiponectin genotypes, were at 59% and 80% higher risk of developing breast cancer, respectively. AdipoR1 rs7539542 (+10225 C → G) CC and CG genotypes were also predicted to carry a lower breast cancer risk. 

In a Chinese case control study, prostate cancer patients exhibited significantly lower levels of adiponectin in circulation. To demonstrate the role of adiponectin in prostate cancer incidence and progression they developed stable transfects of prostate cancer cell lines deficient for adiponectin receptor. Adiponectin administration to parent cell line suppressed cell migration, tube formation, and induced cell cycle arrest, while adiponectin deficiency enhanced the proliferative, migratory, and pro-angiogenic potential of these cells [121]. Gao et al. [122] in 2015 demonstrated that adiponectin overexpression in prostate cancer cells results in depletion of VEGFA and vice versa via an AMPK/TSC2 mediated mechanism. Recently, Shrestha et al. demonstrated the critical role of transcription factor FoxO3A in adiponectin-mediated growth arrest and apoptosis in cancer cells. Globular adiponectin induces p27 but inhibits Cyclin D1 in breast cancer cell lines MCF7 and hepatic cancer cell line HepG2 along with caspase 3/7 activation and FasL expression. Silencing FoxO3A using siRNA inhibited p27 and activated CyclinD1 while preventing caspase and FasL activation suggesting FoxO3A-mediated growth arrest in these cells. On silencing AMPK, however, they observed an inhibition of nuclear translocation of FoxO3A along with inhibition of adiponectin-induced cell cycle arrest and apoptosis. AMPK, thus, acts upstream of FoxO3A in regulating adiponectin cytotoxicity in cancer cells [123]. Another study using HepG2 and Huh7 cell lines elaborates adiponectin-induced inhibition of hepatocellular carcinoma through JNK and mTOR pathway modulation, though upstream regulation remains to be determined. Adiponectin-induced cell death in these cell lines is accompanied by intracellular reactive oxygen species (ROS) accumulation and adiponectin’s effects were inhibited by N-acetylcysteine. Levels of thioredoxin proteins, Trx1 and 2 were altered while overexpression of either of the proteins rescued adiponectin’s effect [124]. 

High circulating adiponectin has also been shown to be associated with 50% lower risk of endometrial cancer irrespective of BMI, hence, it can be considered an independent predictor of endometrial cancer risk. Similarly, adiponectin receptors have been detected in prostate cancer cell lines and in patients. Adiponectin in circulation was evaluated to be negatively correlated with histological grade prostate cancer. Consistently, full-length adiponectin also inhibited growth of prostate cancer cell lines in vitro. In models of CRC, adiponectin knockdown resulted in increased multiplicity of colorectal polyps which were also more aggressive and metastatic with higher COX2 levels compared to their wild-type counterparts suggesting that higher levels of circulating adiponectin could be associated with better prognosis of colorectal cancer as well. In adiponectin-deficient mice, adiponectin inhibited tumor progression and angiogenesis when fed an obesogenic diet but not with normal diet [125,126,127]. Adiponectin deficiency also aggravated azoxymethane-induced (carcinogen-induced) colon cancer in C57BL/6J mice [131]. A series of studies by Saxena et al. demonstrated that adiponectin conferred protection against inflammation-induced colon cancers by preventing apoptosis in the goblet cells and promoting differentiation of epithelial cells to goblet cells [132,133]. In HCT116, HT29, and LoVo CRC cell lines, adiponectin induces G1/S cell cycle arrest with concurrent overexpression of p21 and p27 via AMPK phosphorylation; inhibition of adiponectin receptors freed the cells of adiponectin-induced growth arrest [134]. Moreover, adiponectin rs266729 (−11365 C → G) GG and GC genotypes have been reported to be at 27% lower risk of encountering colorectal cancer compared to individuals with CC genotype, though results in this regard have been inconsistent [135]. 

### 8.3. Inhibition of Invasion, Migration, and Metastasis

Owing to its strong negative association with multiple cancers and its role in tumor angiogenesis and vasculature development, many research groups have studied the involvement of adiponectin in cancer invasion and metastasis. However, not many studies have specifically examined the role of adiponectin in cancer metastasis. Adipokine leptin is a strong predictor of poor outcome in breast cancer. Adiponectin has been shown to counteract the effect of leptin by inhibiting leptin-induced migration and invasion in breast cancer in addition to leptin-induced clonogenicity and anchorage independent cell growth. Adiponectin pretreatment suppresses leptin-induced ERK and Akt signaling. Additionally, it amplifies the protein tyrosine phosphatase 1B (PTP1B) expression and activity, physiological leptin inhibitor and PTP1B inhibition restores leptin activity. Adenoviral adiponectin treatment retards tumor progression in xenograft [136]. In endometrial cancer cell line SPEC-2, adiponectin reverses its metastatic phenotype. Adiponectin inhibits leptin-induced proliferation as well as invasion potential of the SPEC2 cells. Mechanistically, adiponectin prevents leptin-induced invasion by inhibiting signal transducer and activator of transcription 3 (STAT3) phosphorylation and MAPK-mediated nuclear translocation [137]. In liver cancer xenografts, adiponectin inhibits tumor progression and reduces lung metastasis. Adiponectin inhibits hepatic stellate cell activation, intratumoral macrophage infiltration, and diminishes tumor vascularization by downregulating ROCK/IP10/VEGF signaling and inhibition of lamellipodia formation [138]. In non-small cell lung carcinoma (NSCLC), adiponectin prevents migration and invasion of cancer cells by inhibiting epithelial-to-mesenchymal transition (EMT). Adiponectin upregulates epithelial marker expression and decreases mesenchymal markers which could be reversed by knocking down Twist, AdipoR1, and AdipoR2 [139]. Though compelling experimental evidence support metastasis inhibitory effects of adiponectin, results across studies are still inconsistent and more detailed investigation is warranted. 

## 9. Molecular Mechanisms Mediating Adiponectin’s Effects in Cancer

**Figure 2:**
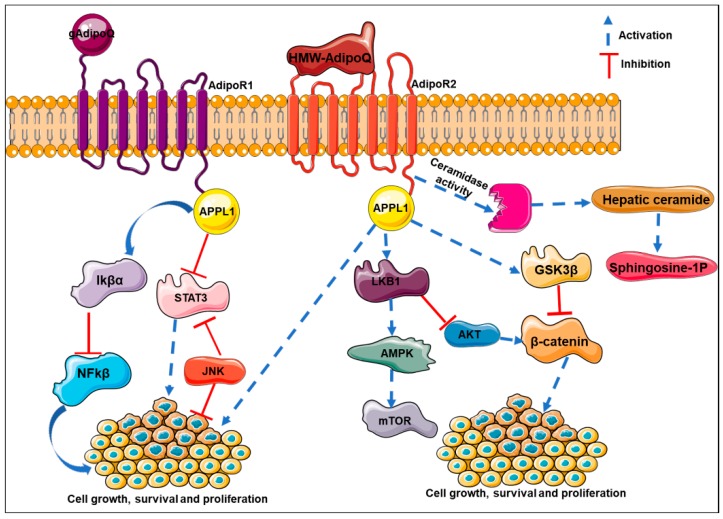
Adiponectin modulates various signaling mechanisms to inhibit caner growth.

The literature clearly suggests that adiponectin can activate several pathways like AMPK, MAPK and PI3K/AKt. AMPK affects cell growth via mammalian target of rapamycin (mTOR), thereby inhibiting the induction of tumor formation. Adiponectin induces growth arrest and apoptosis by activating AMPK in various cell lines in a p53 and p21 dependent manner. In vitro studies of adiponectin on several colon cancer cell lines (HCT116, HT29, and LoVo) show that adiponectin inhibits colon cancer cell proliferation and impairs the cell cycle at G1/S transition phase by inducing p21 and p27 [134]. Adiponectin induces the tumor suppressor gene, *LKB1*, thereby resulting in AMPK activation and inhibition of cell adhesion, invasion and migration in breast cancer cell lines [140,141]. Adiponectin-mediated LKB1 upregulation is also involved in the induction of cytotoxic autophagy leading to tumor inhibition [142]. The role of adiponectin on MAPK signaling remains debatable. The study by Daniele et al. reveal higher adiponectin in the serum samples of chronic obstructive pulmonary disease (COPD) patients compared to control subjects [143]. Adiponectin treatment downregulates ERK1/2 signaling leading to reduction of cell viability in breast and endometrial cancer cell lines [144,145]. Adiponectin-treated MCF7 cells reveal a decrease in the expression level of c-myc, cyclin-D1, and Bcl2 with an increased expression of p52 and Bax, thereby leading to cell cycle arrest [145]. JNK, a member of MAP kinases, has a role in tumor development by regulating cell proliferation and apoptosis [146]. It is also involved in obesity and insulin resistance [146,147]. Similarly, STAT3 (signal transducer and activator of transcription 3) is also involved in cell survival and proliferation and the deregulation of STAT3 leads to tumor progression and metastasis. Saxena et al. reported that adiponectin treatment enhances JNK activation and causes apoptosis in hepatocellular carcinoma cell line in a caspase-3 dependent manner [148]. Reports reveal that adiponectin treatment reduces STAT3 and Akt phosphorylation in liver and prostate cancer cell lines [149]. Adiponectin-treated breast and colorectal cancer cell lines reveal a decrease in PI3K and Akt phosphorylation [150]. At the same time, adiponectin also induces AMPK and inhibits mammalian target of rapamycin (mTOR) cascade in colorectal cancer cell lines [151]. The Wnt signaling pathway plays a proven role in self-renewal and differentiation in different cancer models. The binding of WNT ligand to frizzled activates the signaling cascade by inhibiting glycogen synthase kinase 3 beta (GSK-3β) which is a negative regulator of β-Catenin. The inhibition of GSK-3β promotes the nuclear translocation of β-Catenin, thus, activating WNT signaling. Wang et al. reported that adiponectin inhibits GSK-3β phosphorylation and prevents β-Catenin nuclear translocation in MDA-MB-231 triple-negative breast cancer cells [129]. Liu et al. showed that adiponectin treatment induces Wnt inhibitory factor 1 (WIF1) in a time-dependent manner and results in the decrease of cell proliferation, nuclear translocation of β-Catenin, and reduces expression of cyclin-D1 in breast cancer cells [152]. One of the major anti-apoptotic pathways is the NF-Kβ pathway. Ouchi et al. reported that human aortic endothelial cells pre-incubated with adiponectin show reduced phosphorylation of TNF-alpha-induced Ikappaβ-α, thereby suppressing NFKβ activation via cAMP accumulation. This effect is blocked in the presence of adenylate cyclase or protein kinase A (PKA) inhibitor [153]. Adiponectin modulates various signaling mechanisms to inhibit cancer growth and progression (Figure 2). 

## 10. Potential Therapeutic Modulation of Adiponectin 

Obesity and metabolic syndrome have grown to be the root cause of most life-threatening diseases ranging from type 2 diabetes, cardiovascular diseases, and cancer. Obesity leads to hormonal dysregulation and insulin resistance which initiates a cascade of events leading to failure of the metabolic machinery of the body, hence morbidity. Therapeutic regulation of adiponectin may be achieved either by administration of exogenous recombinant adiponectin or using pharmacological agents to induce increased production of exogenous adiponectin [36]. However, similar to most biologics, mass production of functional adiponectin is challenging since within the biological system it is under intense post-transcriptional and post-translational modifications which are hard to mimic in vitro [36,154]. Bacterial systems lack mammalian protein synthesis machinery and fail to produce functionally active adiponectin. Exploitation of the mammalian culture system for mass production is not a scalable process. In addition, adiponectin has a short half-life in circulation making exogenous administration of recombinant adiponectin a non-feasible approach [36,155]. The only practical mode of adiponectin therapy is, therefore, to induce increased production of endogenous adiponectin using either natural means or pharmacological interventions. The most natural means of boosting adiponectin production is weight loss since adiponectin is the hormone secreted by the lean adipose tissue and is suppressed by leptin and other inflammatory cytokines produced by obese adipose. Multiple effective interventions of weight loss, discussed later, have been strategized in recent years but weight loss remains to be a difficult hurdle. The most feasible method of adiponectin therapy would therefore be the use of pharmacological intervention to enhance adiponectin biosynthesis, bioavailability and bioactivity. The key to designing adiponectin enhancing therapies is to understand its transcriptional and translational regulation. The adiponectin promoter is known to bind a number of transcription factors capable of modulating its activity [30]. It is composed of a PPAR responsive element [19], a CCAAT box [156], multiple C/EBPα enhancers and a sterol regulator element or SRE [157]. Several pharmacological agents have been developed to target or modulate adiponectin machinery. 

### 10.1. Pharmacological Agents

Though results from clinical trials are conflicting, statins including pravastatin [158], simvastatin [159], rosuvastatin [160], and atorvastatin [161] have been reported to be effective in increasing circulating adiponectin. Statins function by releasing cellular oxidative stress resulting in increased multimerization and release. These include ramipril [162], Quinapril [163], Losartan [164], Telmisartan [164,165], Irbesartan [165,166], and Candesartan [166], all of which have shown promising results in clinical trials. They function by enhancing adiponectin secretion via PPARγ, though some are also known to induce transcription. Pioglitazone and Rosiglitazone are known to enhance circulating adiponectin levels 2–4 fold [167,168]. TZDs function by inducing transcription of adiponectin via PPARγ. They have also been found to enhance secretion of folded adiponectin by inhibiting ERp44 and upregulating Ero1-La and DsbA-L. Other potential drugs include non-statin anti-hyperlipidemic drugs like Fenofibrate and Zetia, non-TZD anti-diabetic drugs, such as Acarbose [36] and the sulfonylurea Glimepiride [169] and Sulfonylureas. Androgen blockers have also been proven to be effective at increasing HMW adiponectin and can be used in cases of prostate cancers. 

### 10.2. Weight Loss Interventions

Caloric restriction has been the most commonly implemented intervention for weight loss. It creates an energy deficit forcing the body to utilize energy stored in adipose tissue to fuel basal metabolic activities. However, with the surge in obesity research and better understanding of hormonal regulation of metabolic processes, the therapeutic significance of caloric restriction has been questioned. It has been observed that with constant intake of low energy food, the body activates a coping mechanism by lowering the basal metabolic rate and develops resistance to fat catabolism. As a result, the metabolic processes do not switch, they merely slow down; consequently, there is no change in hormonal milieu of the body. In addition, only recently have we started to fully appreciate the hormone central to all metabolic processes, insulin. It is now believed that an obesogenic diet is one with higher glycemic index rather than caloric density and insulin responds to a spike in blood glucose by directing cells to store energy in the form of fat. The glycemic index of food is determined by its macronutrient composition, precisely the ratio of carbohydrates, fat, protein, and fiber (a form of complex carbohydrate). While caloric restriction may promote some weight loss, it does not seem to have therapeutic benefit. 

In recent years, intermittent fasting and ketogenic diets have been shown to have immense health benefits and have also been utilized as therapeutic regimens. Intermittent fasting refers to a form of diet when food is consumed within small windows of time of a few hours followed by long hours of fasting without calorie restriction. As a result, insulin levels remain constant for long time intervals with a limited period of insulin spike preventing fat storage. During periods of fasting, fat metabolism is induced resulting in weight loss. This mode of feeding has been shown to improve metabolic functions, improve insulin sensitivity, and restore hormonal balance. A recent study suggests that intermittent fasting induces beiging of white adipose tissue via microbiome modulation [170], such change could probably induce adiponectin synthesis and lower leptin levels. Ketogenic diet, on the other hand, relies on dietary fat as a major energy source. It is composed of 60–80% fat, 20% proteins, and only 5–10% carbohydrates most of which is dietary fiber. It works on the principle of insulin response; the glycemic index of macronutrients varies in the order of carbohydrates (minus fibers) > protein > fat. Since fats are the main energy source, insulin response to the diet is minimal, shutting down the process of fat storage. Utilizing fat as the source of energy, the body adapts to fat utilization rather than depending on glycogen stores in hours of need, thus it results in fat loss. Fats and proteins are known to induce high levels of satiety resulting in appetite reduction which also promotes weight loss. A combination of intermittent fasting and a ketogenic diet has been shown to be most effective in promoting weight loss and rewiring metabolic dysfunctions. Body fat loss and increased insulin sensitivity are the most effective and natural methods of reversing obesity-associated inflammation and leptin downregulation, and thus, adiponectin upregulation. Physical activity and exercising is another reliable method of weight loss and is known to reduce inflammation [171]. A recent meta-analysis comprising 2996 individuals investigated metabolic regulation in diabetes patients and how it is affected by exercise [172]. Of all exercise modalities, only aerobic exercise was found to increase adiponectin and decrease leptin levels [172]. Collectively, these studies indicate that combination regimens of diet, exercise, and fasting can help boost adiponectin levels in hypoadiponectinemia. 

## 11. Conclusions

Adiponectin, an important adipocytokine mainly produced by lean adipocytes, is considered a guardian angel adipocytokine owing to its protective functions against various disease states associated with obesity. Adiponectin inversely correlates with obesity and is under tight regulation at transcriptional and translational levels. Though important in most chronic diseases including CVD and T2DM, the role of adiponectin in cancers is most critical. Women who are genetically wired to have lower levels of circulating adiponectin live with a significantly higher risk of breast cancer irrespective of BMI and adiposity. Similar associations have also been observed in other cancers including CRC, prostrate, and hepatic malignancies. Modulation of adipose-secreted hormones regulates metabolic functions of the body, and therefore have direct consequences on cancers which survive by hijacking host metabolic machinery. However, as detailed in this review, adiponectin works in concert with other important hormones including insulin, leptin, and various cytokines making its pharmacological exploitation more difficult. Various strategies have been developed to modulate adiponectin levels in disease state to harness its beneficial effects including pharmacological agents functioning at transcriptional and post-translational levels as well as weight loss strategies. Evidently, adiponectin intervention alone is not sufficient to confront these chronic conditions; it definitely plays an important supportive role in these pathologic states and deserves attention. While pharmacological interventions can prove helpful in treatment of patients genetically deficient in adiponectin, weight management strategies including aerobic exercise and ketogenic diets could be effective in conjunction with other systemic therapies and medications. 

## Figures and Tables

**Table 1:** Obesity-related diseases associated with hypoadiponectinemia.

Diseases	Findings
Hypertension	Obese patients suffering from hypertension display lower adiponectin.
Atherosclerosis	Higher incidences of cardiovascular events are associated with lower hypoadiponectinemia.
Obstructive sleep apnea syndrome	OSAS (Obstructive sleep apnea syndrome) patients revealed lower expression level of adiponectin compared to control patients.
Diabetic retinopathy	T2DM patients with diabetic retinopathy have lower levels of adiponectin compared to T2DM patients without diabetic retinopathy.
Cancer	Multiple evidences suggest low adiponectin levels are associated with the threat of developing several types of cancers.
Metabolic syndrome	Metabolic syndrome represents a group of complications like obesity, hypertension, dyslipidemia, hyperglycemia, and insulin resistance. Enhancement of metabolic syndrome components is associated with a decrease in adiponectin concentration in plasma [88].
Dyslipidemia	Disorder of lipid metabolism leading to high levels of LDL, serum triglycerides, and decreased levels of HDL. Inverse association exists between adiponectin level with LDL and serum triglycerides with a positive association with HDL levels [89].
Hepatic disease non-alcoholic fat	Inverse association exists between adiponectin level in liver with non-alcoholic fatty liver disease as well as non-alcoholic steatohepatitis [90].

**Table 2:** Studies showing circulating HMW adiponectin and its association with cancer risk. RIA: radioimmunoassay; ELISA: enzyme linked immunosorbent assay.

Cancer Type	Ethnicity	Sample	Cases/Control	Method	Reference
Breast cancer	Caucasian	Serum sample	74/76	RIA	[120]
Liver cancer	Asian	Serum sample	59/334	ELISA	[121]
Liver cancer	Asian	Serum sample	97/97	ELISA	[122]
Colorectal cancer	Asian	Plasma sample	165/102	ELISA	[123]
Colorectal cancer	Caucasian	Serum sample	1206/1206	ELISA	[124]
Multiple myeloma	Caucasian	Plasma sample	174/348	ELISA	[125]
Endometrial cancer	Caucasian	Serum sample	62/124	ELISA	[126]
Breast cancer	Asian	Serum sample	66/66	Other method	[127]

## Abbreviations

Acrp30	Adipocyte complement related protein of 30 kDa
apM1	Adipose most abundant gene transcript1
GBP28	Gelatin-binding protein of 28 kDa
AdipoR1	Adiponectin receptor 1
AdipoR2	Adiponectin receptor 2
PPARγ	Peroxisome proliferator-activated receptor gamma
TZD	Thiazolidinedione
PPRE	PPAR response element
HMW adiponectin	High molecular weight adiponectin
FoxO1	Forkhead box protein O1
Sirt1	Sirtuin 1
C/EBPα	CCAAT-enhancer-binding proteins
SERBP	Sterol regulatory element-binding proteins
SRE	SERBP response element
CREB	cAMP response element-binding protein
ATF3	Activating transcription factor 3
NFAT	Nuclear factor of activated T-cells
AP-2β	Activating enhancer binding protein-2β
IGFBP-3	IGF-1-binding protein 3
Id3	Inhibitor of differentiation-3
HIF1α	Hypoxia inducible factor alpha
TNFα	Tumor necrosis factor alpha
IFNγ	Interferon gamma
IL	Interleukin
JNK	c-Jun N-terminal kinases
ERK	Extracellular-signal-regulated kinase
MAPK	Mitogen-activated protein kinases
ERp44	ER chaperone protein 44
Ero-1La	ERO1-like protein alpha
DsbA-L	Disulfide-bond A oxidoreductase-like protein
COX2	Cyclooxygenase 2
VEGF	Vascular endothelial growth factor
PGES	Prostaglandin E synthase
hCG	Human chorionic gonadotropin
AMPK	AMP activated protein kinase
PCOS	Polycystic ovary syndrome
SNP	Single nucleotide polymorphism
GLUT4	Glucose transporter type 4
CNS	Central nervous system
GH	Growth hormone
LH	Luteinizing hormone
eNOS	Endothelial NOS
NFκB	Nuclear factor kappa-light-chain-enhancer of activated B cells
MMP	Matrix metalloproteinase
CVD	Cardiovascular disease
EP4	Prostaglandin E2 receptor 4
SphK	Sphingosine kinase
VCAM-1	Vascular cell adhesion protein 1
BMI	Body mass index
LDL	Low density lipoprotein
HDL	High density lipoprotein
apoA-I	Apolipoprotein A-I
NO	Nitric oxide
PI3K	Phosphoinositide 3-kinase
PDGF	Platelet-derived growth factor
HB-EGF	Heparin-binding epidermal growth factor
BFGF	Basic fibroblast growth factor
EGF	Epidermal growth factor
OSAS	Obstructive sleep apnea syndrome
T2DM	Type 2 diabetes mellitus
IARC	International agency for research on cancer
WCRF	World cancer research fund
SMD	Standard mean differences
RIA	Radioimmunoassay
ELISA	Enzyme linked immunosorbent assay
HGF	Hepatocyte growth factor
bFGF	Basic fibroblast growth factor
FGF-2	Fibroblast growth factor-2
HUVEC	Human Umbilical Vein Endothelial Cells
mTOR	Mammalian target of rapamycin
ROS	Reactive oxygen species
Trx	Thioredoxin
CRC	Colorectal cancer
PTP1B	Protein tyrosine phosphatase 1B
NSCLC	Non-small cell lung carcinoma
EMT	Epithelial to mesenchymal transition
PKA	Protein kinase A
IGF1	Insulin like growth factor-1
COPD	Chronic Obstructive Pulmonary Disease
STAT3	Signal transducer and activator of transcription 3
GSK3β	Glycogen synthase kinase 3 beta
DsbA-L	Disulfide-bond A oxidoreductase-like protein
